# Intrauterine Tampon Identified on CT as an Intrauterine Foreign Body Associated With Bilateral Leg Edema: A Case Report

**DOI:** 10.1155/crog/1505612

**Published:** 2026-04-07

**Authors:** Tadasu Nagaoka

**Affiliations:** ^1^ Department of Diabetes and Endocrinology, Public Central Hospital of Matto Ishikawa, Hakusan, 924-8588, Japan, mattohp.jp

**Keywords:** CARE guidelines, computed tomography, intrauterine foreign body, intrauterine tampon, pelvic congestion, pelvic pain, venous compression

## Abstract

**Background:**

Foreign bodies retained within the vaginal canal are relatively common in gynecological practice, particularly forgotten vaginal tampons, which are well known to cause localized pelvic infection and life‐threatening toxic shock syndrome. However, the transcervical intrauterine migration and retention of a tampon is exceptionally rare, as the cervical canal typically acts as a robust mechanical barrier.

**Case Presentation:**

I report the case of a 32‐year‐old multiparous woman presenting with lower abdominal pain and acute bilateral leg edema. Due to the presence of systemic edema, computed tomography (CT) was utilized as the primary imaging modality to rule out vascular emergencies. The CT scan revealed a hyperdense intrauterine foreign body, later confirmed via speculum examination to be a tampon.

**Conclusion:**

This case emphasizes the importance of considering retained foreign bodies in reproductive‐aged women with unexplained pelvic symptoms and highlights the invaluable role of cross‐sectional imaging in diagnosis when atypical systemic symptoms, such as venous compression resulting in edema, divert initial clinical suspicion. Adherence to the CARE guidelines for clinical case reporting was maintained throughout this manuscript.

## 1. Introduction

Foreign bodies retained within the vaginal canal are relatively frequent presentations in emergency and gynecological settings, with forgotten vaginal tampons ranking among the most common examples [1, 2]. These items are almost exclusively confined to the vaginal vault and are highly associated with localized vaginitis, malodorous purulent discharge, and the risk of staphylococcal toxic shock syndrome mediated by the TSST‐1 superantigen [3].

In stark contrast, the true intrauterine retention of a tampon is exceedingly rare, with only a handful of isolated reports existing in the medical literature [4, 5]. Under normal physiological conditions, the dense fibromuscular stroma of the cervix and the internal os act as a formidable mechanical barrier against the upward migration of intravaginal objects. Therefore, an intrauterine location requires a specific confluence of anatomical alterations and situational vulnerabilities to permit traversal. Early recognition of such translocated foreign bodies is critical to avoid delayed diagnosis, chronic pelvic inflammatory disease, infertility secondary to synechiae, and severe systemic infectious complications.

## 2. Case Presentation

A 32‐year‐old woman, gravida 2, para 2 (G2P2), presented to the emergency department reporting a 3‐day history of escalating lower abdominal pain and progressive bilateral leg edema. She denied any systemic symptoms of fever or nausea and reported no abnormal vaginal discharge. Her medical history was unremarkable, with no recent gynecological surgical interventions or known coagulopathies. She noted that her last menstrual period had begun 6 days prior to her presentation at the hospital.

On arrival, her vital signs were entirely stable and within normal limits, ruling out acute hemodynamic shock. Physical examination demonstrated mild suprapubic tenderness upon deep palpation and significant, pitting dependent edema extending to the mid‐calf bilaterally. An initial external visual inspection of the genitalia noted no abnormal discharge or lesions. A comprehensive internal speculum examination was intentionally deferred at this initial triage stage; the prominent presentation of acute bilateral leg edema raised a high clinical suspicion for a vascular emergency, such as deep vein thrombosis (DVT) extending into the pelvis, or extrinsic venous compression by an occult mass, which took diagnostic precedence.

Comprehensive laboratory results, including complete blood count, inflammatory markers (C‐reactive protein), and coagulation profiles (D‐dimer), were within normal limits. Urinalysis was negative for infection or hematuria.

**Figure 1:**
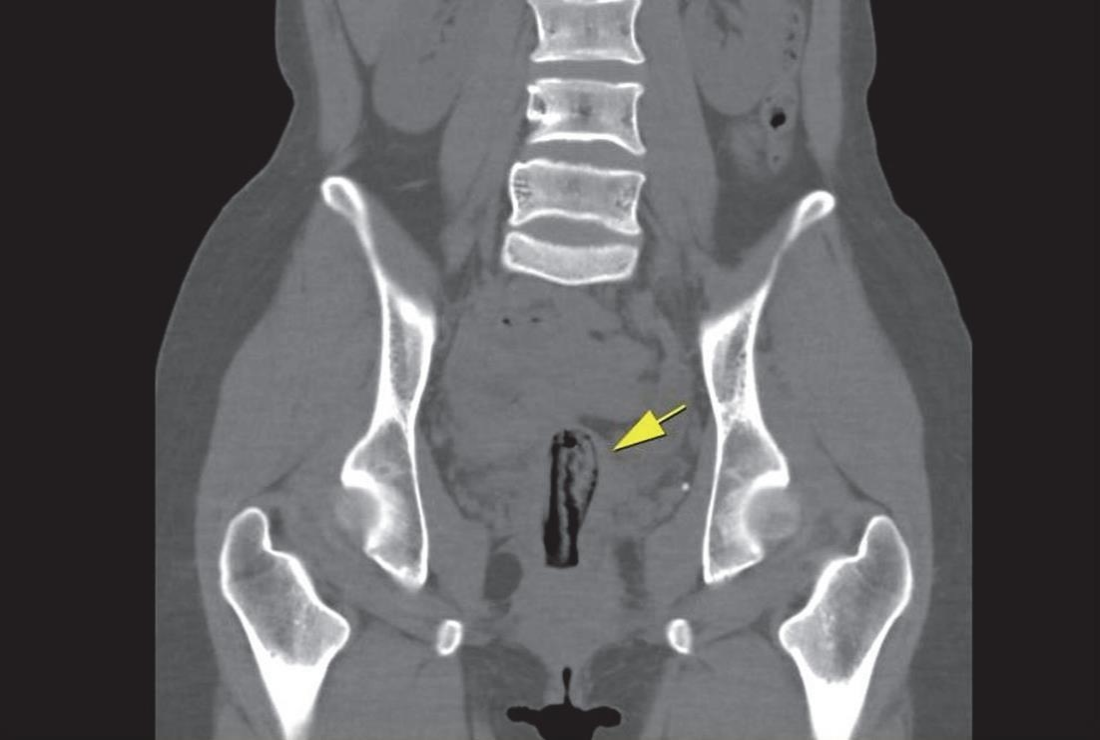
Coronal abdominal CT scan showing a linear high‐density (hyperdense) structure centrally located within the uterine cavity (yellow arrow), subsequently confirmed via pelvic examination to be an intrauterine tampon.

An abdominopelvic computed tomography (CT) scan with intravenous contrast was performed prior to any gynecological ultrasound. This modality was selected specifically to rapidly evaluate the retroperitoneal space, the inferior vena cava, and the bilateral iliac veins for possible thrombosis or malignant compression. The CT scan successfully ruled out DVT but unexpectedly revealed a linear, high‐density (hyperdense) structure centrally located within the endometrial cavity of the uterus (Figure 1). Due to its high attenuation, this structure was initially suspected to be a migrated metallic or plastic intrauterine contraceptive device (IUD); however, upon questioning, the patient vehemently denied ever having such a device inserted.

Further detailed medical interviewing revealed a history of tampon insertion during a period of profound alcohol intoxication several days earlier, with the patient having absolutely no recollection of its subsequent removal. Prompted by the specific CT findings and the new historical context, a targeted speculum examination was finally conducted. During this examination, the string of a menstrual tampon was clearly visualized protruding downward from the external cervical os. The intrauterine tampon was subsequently grasped firmly with sponge forceps and extracted manually under direct visualization via the vagina. The extraction was completed without complication and required no cervical dilation.

Symptoms improved promptly following the removal of the infectious nidus. Crucially, both the lower abdominal pelvic pain and the prominent bilateral leg edema completely resolved within 48 h of the extraction. The patient was discharged home safely without the need for prolonged antibiotic therapy or surgical debridement.

## 3. Discussion

Retained tampons are overwhelmingly located within the vaginal vault and may present clinically with foul‐smelling discharge, localized infection, or toxic shock syndrome [1–3]. True intrauterine retention is exceedingly rare, with isolated reports in the literature mostly relating to rigid devices rather than soft tampons [4, 5].

The biomechanical mechanism by which a highly compressible tampon could traverse the rigid cervical canal in this specific case can be explained by a triad of compounding factors. First, the patient’s multiparous status (G2P2) fundamentally alters cervical architecture, often leaving the external and internal os more patulous than in a nulliparous state. Second, the incident occurred during active menstruation (day 3 of her cycle), a physiological phase characterized by prostaglandin‐mediated cervical dilation and reduced mucus viscosity. Finally, the patient’s state of severe alcohol intoxication likely severely impaired her proprioception and pain perception, resulting in the undirected, forceful insertion of the tampon beyond the posterior vaginal fornix and directly into the lower uterine segment [6].

Furthermore, the presentation of bilateral leg edema in this patient is highly unusual for a retained foreign body, yet it is pathophysiologically sound when considering pelvic hemodynamics. I hypothesize that the retained intrauterine tampon acted as a potent nidus for severe localized pelvic inflammatory disease. This intense foreign body reaction induced pronounced uterine hyperemia, tissue edema, and overall organ swelling. The engorged uterus, acting temporarily as a massive inflammatory pelvic mass, exerted transient extrinsic mechanical compression on the bilateral common iliac veins against the pelvic sidewall. This mechanical obstruction of the pelvic venous outflow tract increased hydrostatic capillary pressure in the lower extremities, overcoming plasma oncotic pressure and manifesting as bilateral dependent edema. The rapid, complete resolution of the edema within 48 h of tampon removal serves as clinical confirmation that the symptom was driven by reversible mechanical compression and localized inflammatory congestion, rather than a systemic vascular pathology or primary lymphatic failure.

This case underscores the critical importance of considering foreign body retention in reproductive‐aged women with unexplained pelvic pain, even when presentations are highly atypical. While transvaginal ultrasound remains the undisputed gold standard for evaluating suspected gynecological pathology, this case demonstrates that cross‐sectional imaging such as CT can be invaluable in identifying intrauterine foreign bodies when gynecological clinical suspicion is initially low, and emergency algorithms rightfully prioritize the exclusion of life‐threatening vascular events. Radiologists must be aware that on CT, a blood‐ and fluid‐soaked tampon loses the negative attenuation of trapped air seen in dry tampons and instead mimics the high‐density radiological appearance of calcified tissue, retained products of conception, or organized hematomas.

## 4. Conclusion

The case displays the successful, simple manual extraction of a rare intrauterine tampon, which highlights the critical importance of comprehensive assessment and considering foreign body retention in reproductive‐aged women with unexplained pelvic pain. Furthermore, it demonstrates that cross‐sectional imaging, such as CT, is invaluable in identifying translocated foreign bodies when clinical presentations are highly atypical, allowing for immediate curative intervention and the prevention of severe secondary complications.

## 5. Learning Points

• Retained menstrual tampons are almost exclusively vaginal; true uterine retention is extremely rare but biomechanically possible under conditions of multiparity, menstrual cervical dilation, and altered patient sensorium [4, 5].
• Foreign body retention must remain on the differential diagnosis for reproductive‐aged women presenting with unexplained pelvic symptoms [2, 3].
• Severe localized pelvic inflammation and uterine engorgement from an intrauterine foreign body can cause extrinsic venous compression of the iliac vessels, presenting atypically as acute bilateral leg edema.
• Advanced cross‐sectional imaging (like CT) is highly useful for detecting intrauterine foreign bodies when clinical symptoms are atypical and diagnostic algorithms prioritize ruling out vascular etiologies [4].
• Prompt recognition and manual removal of the foreign body is curative, rapidly reverses secondary compressive symptoms, and prevents severe infectious complications such as toxic shock syndrome [1, 3, 6].

## Author Contributions

Tadasu Nagaoka was the primary attending physician, collected all clinical data, conducted the literature review, drafted the manuscript, and reviewed and approved the final version.

## Funding

The author formally declares that no financial support, grants, or funding of any kind was received for the clinical care, research, authorship, or publication of this article.

## Ethics Statement

Formal ethical approval was waived by the local Institutional Review Board (IRB) for this retrospective case report, provided that no identifiable patient health information was included in the manuscript or imagery.

## Consent

Written informed consent for publication was not required in this case, as the report includes only anonymized clinical images and highly deidentified historical details from which the patient cannot be recognized.

## Conflicts of Interest

The author declares no conflicts of interest.

## Data Availability

Data sharing is not applicable to this article as no new datasets were generated or analyzed during the current study. The clinical data that support the findings of this case report are strictly restricted from public access in order to protect patient privacy and preserve medical confidentiality, in accordance with institutional ethical guidelines.

## References

[1] Schlievert P. M. and Davis C. C. , Device-Associated Menstrual Toxic Shock Syndrome, Clinical Microbiology Reviews. (2020) 33, no. 3, e00032–e00019, 10.1128/CMR.00032-19. 32461307 PMC7254860

[2] Barber M. D. , Brubaker L. , and Burgio K. L. , et al.Comparison of 2 Transvaginal Surgical Approaches and Perioperative Behavioral Therapy for Apical Vaginal Prolapse, Obstetrical & Gynecological Survey. (2014) 69, no. 7, 371–381, 10.1097/OGX.0000000000000079.

[3] Duhan N. and Sirohiwal D. , Retained Foreign Bodies in the Vagina: A Clinical Study, Journal of the Indian Medical Association. (2010) 108, no. 9, 583–584.21510531

[4] Das S. and Singh S. , Role of Imaging in Evaluation of Intravaginal and Intrauterine Foreign Bodies, The British Journal of Radiology. (2016) 89, no. 1062, 20150968.

[5] Benzoni C. , Gazzola L. , and Bernasconi D. P. , Retained Intravaginal Foreign Body Mimicking Endometrial Carcinoma: Case Report and Literature Review, European Journal of Gynaecological Oncology. (2005) 26, no. 6, 659–660.

[6] Wehmeijer L. M. , Hage J. J. , Eberz B. , and van Beurden M. , Partially Plexiform Neurofibroma of the Labia Minora and Clitoral Hood—A Prognostic Dilemma, Journal of Lower Genital Tract Disease. (2015) 19, no. 3, e55–e57, 10.1097/LGT.0000000000000100, 2-s2.0-84937725706. 25658713

